# Measurement Properties of the Duke Activity Status Index in Arab Patients with Cardiovascular Disease

**DOI:** 10.3390/ijerph192113783

**Published:** 2022-10-23

**Authors:** Ali Albarrati, Abeer H. Abdulghani, Monira I. Aldhahi, Rakan Nazer

**Affiliations:** 1 Rehabilitation Sciences Department, College of Applied Medical Sciences, King Saud University, P.O. Box 10219, Riyadh 11433, Saudi Arabia; 2 Department of Rehabilitation Sciences, College of Health and Rehabilitation Sciences, Princess Nourah bint Abdulrahman University, P.O. Box 84428, Riyadh 11671, Saudi Arabia; 3 Cardiac Sciences Department, College of Medicine, King Saud University, P.O. Box 3642, Riyadh 12372, Saudi Arabia

**Keywords:** activity limitation, CVD, DASI, outcome measure, psychometric properties

## Abstract

The aim of the study was to examine the measurement properties of the Arabic version of the Duke Activity Status Index (DASI) in patients with cardiovascular disease (CVD). A sample of 100 Arab patients with CVD completed the Arabic version of the DASI and underwent an exercise stress test (EST) on the first visit, and the metabolic equivalent (MET) was obtained from each outcome measure. On the second visit, patients with CVD completed the Arabic version of the DASI along with the global rating of change scale (GRC). Reliability, including the internal consistency, test–retest reliability, and construct validity, were examined. Patients with CVD (86 males), mean (SD) age 54.98 (10.2) years, completed the study. The Cronbach’s alpha was 0.87, and the intraclass correlation coefficient (ICC_2,1_) was 0.93. The estimated MET and peak VO_2_ obtained from the DASI were correlated with the estimated MET and peak VO_2_ obtained from the EST (r = 0.58, r = 0.56, all *p*-values < 0.001). The Arabic version of the DASI is a simple, quick, reliable, and valid measure of functional capacity in Arabic-speaking patients with CVD. The DASI may serve as a screening tool for functional capacity in patients with CVD in clinical settings.

## 1. Introduction

Cardiovascular disease (CVD) is prevalent and recognized globally as the leading cause of morbidity and mortality and a major contributor to the burden of physical disability [1]. In Saudi Arabia, unhealthy lifestyle behavior is a substantial risk factor for the high prevalence of CVD [2]. Patients with CVD usual adopting a sedentary lifestyle because of their inability to perform functional activities, subsequently worsening their health status [3].

Functional capacity assessment is key in the management of patients with CVD, as it serves as a diagnostic, therapeutic, and prognostic tool to guide and monitor medical and rehabilitation interventions. The gold standard for assessing functional capacity is the cardiopulmonary exercise test (CPET) [4]. It provides a specific measurement of cardiopulmonary and musculoskeletal systems performance [4,5]. However, CPET is only limited to a few tertiary centers and requires a higher level of expertise. Another laboratory-based testing that has evolved in clinical practice as an indirect assessment of cardiorespiratory fitness is exercise stress testing (EST), which can be used for the detection of cardiovascular disease [5]. However, similar to CPET, EST requires a qualified healthcare provider to administer; thus, patients with pre-existing musculoskeletal disorders may experience difficulty performing the test.

The Duke Activity Status Index (DASI) is a patient-reported outcome measure of functional capacity in individuals with CVD [6]. It is a brief 12-item, self-administered questionnaire to determine physical functional status by capturing the patient’s ability to perform 12 common daily living activities, including personal care, ambulation, housework, yard work, sexual relations, and recreational activities, in which each item is weighted according to its known metabolic cost in metabolic equivalent units (MET) [6]. The DASI has been found to predict exercise capacity, determine eligibility for exercise stress testing, and identify patients with CVD with an increased likelihood of inadequate stress test results [7,8,9]. However, there is no study that has examined the measurement properties of the DASI among Arab patients with CVD. Therefore, this study aimed to evaluate the measurement properties of the DASI in Arab patients with CVD.

## 2. Materials and Methods

### 2.1. Setting and Participants

This was a cross-sectional study involving stable adult patients with CVD. In this study, we recruited patients who were eligible to perform the EST from the Prince Sultan Cardiac Center [4]. Patients were excluded if they had respiratory, neurological, or severe musculoskeletal diseases or if they could not read and understand Arabic.

### 2.2. Ethical Approval

The study was approved by the Institutional Review Boards of King Saud University (IRB number: E-21-6320) and Prince Sultan Cardiac Center (IRB number: HP-01-R079). All patients signed a written informed consent before enrollment. The study was carried out according to the principles of the Declaration of Helsinki between January 2022 and April 2022.

### 2.3. Sample Size Estimation

Sample size estimation was based on the consensus-based standards for the selection of health measurement instruments (COSMIN) [10]. COSMIN considered a sample size of 100 patients with CVD an excellent sample size to examine the internal consistency, test–retest reliability, measurement error, and construct validity of the Arabic version of the DASI.

### 2.4. Procedures

#### 2.4.1. Demographic and Clinical Data Assessment

The eligible patients underwent height and weight measurements, and their body mass index (BMI) was calculated and recorded in kg/m^2^. Patients with CVD were interviewed to collect data related to their medical history, and their health status was evaluated during the first visit to the cardiac center. The CVD-related data, including diagnosis, recent left-ventricle ejection fraction within the previous four weeks, medications, and comorbidities, were retrieved from the electronic medical files of the patients.

During the first visit, patients with CVD completed the Arabic version of the DASI and underwent the EST. On the second visit, which took place after two days from the first visit, patients with CVD completed the DASI and the Global Rating of Change Scale.

#### 2.4.2. The Arabic Version of the Duke Activity Status Index (DASI)

Patients with CVD were asked to complete the Arabic version of the DASI. The 12-item DASI included questions about the patient’s ability to perform various daily activities: personal care (one item), ambulation (four items), housework (three items), yard work (one item), sexual relationships (one item), and recreational activities (two items) [6]. The Arabic version of the DASI is reliable and valid in Arab patients with COPD [11]. The patient’s answer was binary: “yes” or “no” for each item. Each item was evaluated in proportion to the metabolic cost of each activity in MET. Assertive answers were summed to obtain the total score, which ranges from 0 to 58.2 points, with the equivalent ranges of 0 to 9.89 METs. The higher the scores, the better the functional capacity.

#### 2.4.3. Exercise Stress Test (EST)

All the patients with CVD were instructed to maintain their usual medication and avoid caffeine, smoking, and moderate-to-vigorous exercise 24 h before the test day. Exercise test procedures, including screening for contraindications and termination criteria, were followed according to the recommendations of the American Heart Association [12]. Each patient was prepared for the test and received a detailed explanation of the testing procedure and the purpose of the test, including the nature of progressive exercise, symptoms and signs of the end points, and possible complications. A cardiologist conducted the EST on a motorized treadmill in accordance with the Bruce protocol. A 12-lead electrocardiography (ECG) was used to monitor the patient before and during the testing and until the end of the recovery period. The achieved MET, the maximum heart rate, blood pressure, the symptoms of exercise, and the reason for the termination were recorded simultaneously for each patient. To avoid confounding factors, the cardiologist conducting the EST was unaware of the DASI score of each patient.

#### 2.4.4. Global Rating of Change Scale (GRC)

The scale was used to assess the stability of each patient’s health status between the two visits. It is a reliable and valid self-reported scale that involves a single question to determine the improvement or deterioration of an individual over a period of time [13]. The current study used an 11-point GRC scale, ranging from 5 (a very great deal better) to −5 points (a very great deal worse). The acceptable change in GRC scores between the two visits was 1 (a little bit better, almost the same) to 1 point (a little bit worse, almost the same), with 0 points indicating no change.

### 2.5. Data Analysis

The data were analyzed using the IBM SPSS Statistics for Windows, Version 27.0 (IBM Corp., Armonk, NY, USA). The data were tested for normality prior to the analyses. Descriptive data were presented as a mean and standard deviation (SD) for parametric data, median (interquartile range) for non-parametric data, or number and percentage of the total sample. A statistical significance level was set at *p* < 0.05.

#### 2.5.1. Reliability

The internal consistency of the Arabic version of the DASI was examined using Cronbach’s alpha [14,15]. In the current study, a Cronbach’s alpha of 0.90 or more was considered an excellent internal consistency. The test–retest reliability of the Arabic version of the DASI was examined using the intra-class correlation coefficient (ICC_2,1_). An ICC value between 0.75 and 0.90 indicated a good reliability, and a value greater than 0.90 was considered excellent [14,15]. The standard error of measurement (SEM) was calculated using the following formula: SEM = SD$\sqrt{1-ICC}$, where SD is the standard deviation of the Arabic version of the DASI scores at baseline [14,15]. The minimum detectable change (MDC_90%_) was calculated using the following formula: MDC_90%_ = 1.65 × $\sqrt{2}$ × SEM [14,15]. The Bland–Altman analysis was used to quantify the difference between the Arabic version of the DASI measurements on the two visits [16].

#### 2.5.2. Validity

The construct validity of the Arabic version of the DASI was examined by using bivariate correlations between the DASI scores and the EST based on the normality test. We hypothesized that the estimated MET and peak VO_2_ from the Arabic version of the DASI would have moderate to strong positive correlations (>0.40) with the estimated MET and peak VO_2_ from the EST.

## 3. Results

### 3.1. Participants Physical and Clinical Characteristics

A total of 112 patients (88% male) were included in this study and completed the first visit. After a mean period of 2 days, 100 patients (86 males) returned for the second visit and completed the Arabic version of the DASI. The remaining (12 participants) were unable to attend due to transportation issues. The physical and clinical characteristics of patients with CVD are described in Table 1. Patients with CVD completed the GRC, and the results demonstrated that they were clinically stable with no changes in their health status.

### 3.2. Floor and Ceiling Effects

The number of patients with the lowest and highest possible scores was one (1%) and eight (8%), respectively, indicating that no floor or ceiling effects were present for the Arabic version of the DASI.

### 3.3. Reliability

The Arabic version of the DASI demonstrated excellent internal consistency, and the Cronbach’s alpha was 0.86. The test–retest reliability was excellent, ICC_2,1_ = 0.93 (95% CI: 0.89 to 0.95, *p* < 0.001). The SEM and MDC_90_ were 3.70 points and 8.63 points, respectively. The limits of agreement between the two DASI measurements found a mean difference of −1.33, with an SD of 7.01. The upper limit was 12 points, and the lower limit was −15 points at a 95% confidence level.

### 3.4. Construct Validity

The total DASI score demonstrated significant positive correlations with the estimated peak VO_2_ obtained from the EST (r = 0.58, *p* < 0.001) and the total EST duration (r = 0.56, *p* < 0.001). Similarly, the estimated MET and peak VO_2_ from the DASI were correlated with the estimated MET and peak VO_2_ obtained from the EST, respectively (r = 0.58, r = 0.56, All *p* < 0.001).

## 4. Discussion

The current study explored the measurement properties of the DASI in Arab patients with CVD. The DASI did not reveal any floor or ceiling issues and had excellent internal consistency, good test–retest reliability, and an acceptable measurement error. In addition, the DASI demonstrated evidence supporting its construct validity as a measure of functional capacity in patients with CVD.

The evaluation of functional capacity is crucial in the management of patients with CVD, as it provides valuable information on the patient’s general health, disease status, and response to interventions [4]. Ample evidence reports that patients with CVD revealed reduced functional capacity, which is a key factor contributing to increased morbidity, hospitalizations, and mortality rates [3]. Therefore, integrating functional capacity assessment as an essential part in the clinical management of patients with CVD is fundamental. Routine evaluation of functional capacity in clinical settings enables healthcare providers to provide appropriate and effective therapeutic interventions and prevent complications related to functional inactivity. Healthcare providers usually have limited time with patients with CVD in clinical settings, making them unable to perform functional capacity assessment. The DASI is a useful functional capacity index that captures several daily activities, including personal care, ambulation, housework, yard work, sexual relations, and recreational activities [6].

The results of the current study indicated that the Arabic version of the DASI demonstrated excellent internal consistency and test–retest reliability consistent with our hypothesis. A similar study by Coutinho-Myrrha et al. [17] showed that the Portuguese version of the DASI among patients with CVD had excellent internal consistency, Cronbach’s α = 0.93, and test–retest reliability with an ICC of 0.87. In parallel, Fan and colleagues [18] reported a similar internal consistency for the DASI, Cronbach’s α = 0.86, in patients with heart failure. On the contrary, the Hindi version of the DASI demonstrated a moderate internal consistency with a Cronbach’s α of 0.65 in patients with myocardial infarction [9]. This discrepancy may be attributed to language, culture, and socioeconomic status variability among Hindi participants. In a recent study in Arab patients with COPD [11], the DASI had similar internal consistency (Cronbach’s α = 0.87) and test–retest reliability (ICC = 0.95).

Furthermore, the measurement error of the DASI in patients with CVD has not been previously established. In the current study, we established the measurement error of the Arabic version of the DASI in patients with CVD using SEM and MDC_90%_. The SEM in our study was 3.70 points, which represents 6.4% of the total DASI range, while the MDC_90%_ was 8.63 points, representing 14.8% of the total DASI score. These measurement error values are considered small and acceptable, as they represent only a small magnitude of the total scale range of the DASI. In the clinical context, a score greater than or equal to 9 on the DASI is clinically considered a true change in the functional capacity of the patient. Similarly, the SEM and MDC_90%_ of the Arabic version of the DASI in patients with COPD were 3.5 and 8.2 points, respectively [11].

The DASI is presumed to measure one construct, which is functional capacity. In the current study, the construct validity of the Arabic version of the DASI was assessed by quantifying the correlation coefficient with the EST. The Arabic version of the DASI was positively correlated with the EST indices, including the peak MET, exercise time, and the estimated peak VO_2_, suggesting that patients with high Arabic DASI scores were more likely to reach a high stage in the EST; therefore, they had a high functional capacity. Previous studies using different versions of the DASI have yielded similar findings between different DASI scores and maximal functional capacity measured by the treadmill EST in patients with different cardiac diseases [16,17].

Furthermore, this study demonstrated that the Arabic version of the DASI had no floor or ceiling effects in patients with CVD, indicating its ability to differentiate between patients with CVD with better and poor functional capacity. Several studies reported that the DASI may serve as a useful tool for risk stratification, determining eligibility for the EST, and guiding clinical decisions [7,8,9,19]. In patients with CVD, the DASI identified patients with an increased likelihood of inadequate stress test results [9]. Other studies found the DASI as a clinically applicable self-assessment tool for estimating functional capacity, predicting long-term prognosis, and determining risk of mortality in patients with heart failure and peripheral vascular disease [8,19]. Therefore, incorporating the DASI into clinical settings can lead to an efficient and effective management by providing the best patient-centered care.

### Limitations

In this study, we examined the construct validity of the DASI against the most common clinical laboratory test administered by cardiologists to assess functional capacity. However, it would have been more robust if we examined the DASI against the gold standard, CPET. Nevertheless, cardiologists do not use CPET practically in our clinical settings, especially among patients at risk of falling, and it requires expertise. In this study, the majority of patients were males, which may not represent the general population with CVD. The DASI needs more testing of measurement properties, especially responsiveness, which is an important quality aspect of assessment tools in research and clinical settings.

## 5. Conclusions

The Arabic version of the DASI demonstrated no floor or ceiling issues and had excellent internal consistency and test–retest reliability and an acceptable measurement error. The DASI revealed evidence supporting its construct validity as a clinically applicable measure of functional capacity in patients with CVD. Due to its simplicity and rapidity, the Arabic version of the DASI can be utilized to quantify functional capacity in Arabic-speaking patients with CVD in routine clinical practice worldwide.

## Figures and Tables

**Table 1:** Physical and clinical characteristics of patients with CVD (*N* = 100).

Variable	Mean ± SD
Male/Female (*N*)	86/14
Age (years)	54.98 ± 10.2
Weight (kg)	84.61 ± 15
Height (cm)	168.4 ± 8.1
BMI (kg/m^2^)	29.83 ± 4.6
**Type of CVD**	
IHD	87 (87%)
Valvular disease	13 (13%)
**Smoking status,** ***N*** **(%)**	
Non-smoker	55 (55%)
Smoker	15 (15%)
Ex-smoker	30 (30%)
EF (%)	49 ± 11
**Functional capacity**	
DASI score	39.89 ± 13.12
DASI VO_2_ (ml/kg/min)	26.47 ± 6.01
Estimated MET from DASI	7.56 ± 1.72
Estimated peak VO_2_ (ml/kg/min)	33.18 ± 2.80
Estimated MET from EST	9.84 ± 3.30

Abbreviations: N, total number of patients; SD, standard deviation; kg, kilogram; BMI, body mass index; CVD, cardiovascular disease; IHD, ischemic heart disease; EF, ejection fraction; DASI, Duke Activity Status Index; VO_2_, oxygen consumption; MET, metabolic equivalent; EST, exercise stress test.

## Data Availability

The data that support the findings of this study are available from the corresponding author, A.B., upon reasonable request.
